# Pulmonary tumors associated with the JC virus T-antigen in a transgenic mouse model

**DOI:** 10.3892/or.2013.2782

**Published:** 2013-10-02

**Authors:** AKIRA NOGUCHI, KEIJI KIKUCHI, TAKASHI OHTSU, MITSUYO YOSHIWARA, YOSHIYASU NAKAMURA, YOHEI MIYAGI, HUACHUAN ZHENG, YASUO TAKANO

**Affiliations:** 1 Kanagawa Cancer Center Research Institute, Yokohama, Kanagawa 241-0815, Japan; 2 Department of Biochemistry and Molecular Biology, Institute of Pathology and Physiology, College of Basic Medicine, China Medical University, Shenyang 110001, P.R. China

**Keywords:** JC virus, T-antigen, pulmonary tumors, transgenic mice

## Abstract

Many attempts to demonstrate the oncogenic role of the JC virus (JCV) have been partially successful in producing brain tumors, either by direct inoculation of JCV into the brain or in transgenic models in rodents. We previously reported the presence of JCV DNA with a relatively high incidence in pulmonary and digestive organs. However, we could not prove the oncogenic role of JCV. We prepared a transgene composed of the K19 promoter, specific to bronchial epithelium with the JCV T-antigen and established transgenic (TG) mice. Pulmonary tumors were detected without any metastasis in 2 out of 15 (13.3%) 16-month-old K19/JCV T-antigen TG mice. Using immunohistochemistry (IHC), these tumors showed JCV T-antigen, p53 and CK 19 expression, but not expression of nuclear and cytoplasmic β-catenin and insulin receptor substrate 1 (IRS1). IHC revealed the same expression pattern as in the bronchial epithelium of the TG mice. One tumor, which was examined with laser capture microdissection and molecular biological tools, demonstrated an EGFR mutation but not a K-ras mutation. We propose that the pulmonary tumors were derived from the JCV T-antigen in a TG mouse model. These findings shed light on pulmonary carcinogenesis.

## Introduction

JC virus (JCV), a member of the polyomavirus genus, which also includes SV40 and BK viruses, is a non-enveloped, 5,130-bp double-stranded DNA virus that forms minichromosomes with cellular histones (1). The JCV early region encodes 2 oncoproteins, large T-antigen (T-Ag) and small t antigen (t-Ag). The late region encodes 3 capsid proteins, VP1, VP2 and VP3, and a small regulatory protein, agnoprotein (2). The early viral proteins have high transformation and oncogenic potential in experimental systems (3). There are 2 possible outcomes of JCV infection. One possible outcome is the support of viral DNA replication by permissive cells, such as oligodendrocytes, resulting in a lytic infection. Another possibility is silent or abortive infection that may be linked to cell transformation in nonpermissive cells, such as those of the pulmonary and digestive epithelium (4).

As JCV has been frequently detected in tumors of the human central nervous system (CNS) (5), it has been suggested that this virus may have a possible association with a variety of human CNS tumors. However, the mechanisms involved in JCV-associated oncogenesis must be clarified since studies provide contradictory evidence as to whether or not JCV plays a causative role in human CNS tumors (6). Laghi *et al*(7), followed by Enam *et al*(8), were the first to report the presence of JCV DNA in colorectal cancers. We evaluated the presence of JCV DNA in Japanese patients with colorectal (9), gastric (10), oral (11) and lung cancer (12). While JCV DNA was detected in cancers of these organs, we could not demonstrate an association between the presence of JCV DNA and its oncogenic role.

Approximately 4 decades have passed since the first report that JCV is found in brain tumors following inoculation of this virus into the brain of newborn hamsters (13). Subsequently, several studies have shown that brain tumors arise in JCV transgenic mouse models (14,15). It is well-known that the JCV receptor is the 5-HT2A serotonin receptor, which is primarily restricted to glial cells (16). Therefore, it may be reasonable to conclude that JCV has a strong tendency to form neurotropic tumors (6). However, since JCV DNA has been shown to be present in pulmonary and digestive organs, we speculate that JCV may infect these organs through an unknown receptor. We created transgenic (TG) mice with a transgene including the K-19 promoter, which is specific to bronchial and digestive epithelium (17) and the JCV T-antigen. We found pulmonary tumors in 2/15 mice (13.3%) with no tumors in any other organs.

## Materials and methods

### Establishment and generation of K-19/JCV T-antigen transgenic mice

TG mice were generated using a 3.2-kb *Bal*I/*Nci*I restriction fragment from the plasmid pBJC, containing the K-19 promoter (a kind gift from Professor H. Oshima, Kanazawa University Cancer Research Institute) and the coding region for the viral early genes, large T- and small t-antigens. The DNA was microinjected into the pronucleus of fertilized mouse oocytes generated by the mating of C57BL/6J mice (Chrysalis DNX, Princeton, NJ, USA). Founder animals were mated with C57BL/6J mice, and all mice were screened for the presence of the transgene by DNA extraction from the tail tissue and real-time PCR analysis with primers specific to the transgene. Real-time quantitative PCR was performed using the SYBR^®^ Premix DimerEraser (Takara Bio, Inc., Ohtsu, Japan) kit and LightCycler^®^ (Roche Applied Science, Indianapolis, IN, USA). The primer set (5′-TGCCACTGTCTATTGGCCCCT-3′ and 5′-TTGGGGCACATGGCAATGCTGT-3′) was designed to detect JCV T-antigen DNA (amplicon size 168 bp), and reaction mixtures were prepared according to the manufacturer’s protocol. After amplification, using hemizygous and wild-type mice (C57BL/6J), we generated standard curves. The homozygous, hemizygous and non-TG states in the target DNA samples were clearly distinguished by the difference in these C_t_ values. Potentially homozygous animals were validated by test breeding with wild-type partners. Pure JCV T-antigen^+/+^ mice were achieved after 3 generations.

In the present study, 15 TG mice (8 males and 7 females) and 10 control mice (5 males and 5 females) were euthanized by CO_2_ inhalation at 16 months of age and were autopsied systemically.

### Histologic and immunohistochemical (IHC) analyses of TG mice

Tissues from the lung, heart, liver, pancreas, spleen, kidney, esophagus, stomach, small intestine, large intestine and brain were obtained from TG and C57BL/6J control mice and fixed in 10% formalin. Histological examination was carried out by 3 pathologists (A.N., Y.M. and Y.T.).

The IHC procedure was similar to that described previously (12). Primary antibodies were directed against JCV T-antigen (1:100 dilution, PAb416, mouse mAb; EMD Millipore, Darmstadt, Germany), p53 (1:50, PAb122, mouse mAb; Enzo Life Science, Farmingdale, NY, USA), β-catenin (1:1,000, rabbit pAb; Cell Signaling Technology, Inc., Beverly, MA, USA), IRS-1 (1:50, sc-8038, mouse mAb; Santa Cruz Biotechnology, Inc., Santa Cruz, CA, USA), cytokeratin CK-19 (1:100, rabbit mAb; Epitomics, Burlingame, CA, USA), CK-7 (1:100, KRT7, rabbit pAb; Proteintech Group, Chicago, IL, USA), CK-20 (1:50, mouse mAb; GeneTex, San Antonio, TX, USA), CD3 (1:100, goat pAb, Santa Cruz Biotechnology, Inc.), CD4 (1:50, mouse mAb; EMD Millipore), CD5 (1:50, mouse mAb; Abnova, Taipei, Taiwan), CD8 (1:50, rabbit mAb; GeneTex), CD20 (1:50, rabbit mAb; EMD Millipore), CD21 (1:50, mouse mAb; Santa Cruz Biotechnology, Inc.) and CD79a (1:100, rabbit pAb; LifeSpan Biosciences, Seattle, WA, USA).

### Mutation analysis of K-ras and epidermal growth factor receptor

DNA samples from tumor lesions and bronchial epithelium were selectively captured from 10 μm dewaxed sections using a laser capture microdissection (LCM) system (LM200, Olympus, Tokyo). Isolation of DNA from LCM specimens was performed with a PicoPure^®^ DNA Extraction kit (Arcturus Engineering, Mountain View, CA, USA) according to the manufacturer’s protocol. The primers used for detection of codon 12 mutations were described elsewhere (18). The primers used for detection of epidermal growth factor receptor (EGFR) exon 18–21 mutations were 18S:2349–2369, 5′-TCGTGGAACCTCTCACACCCA-3′ and 18A:2467–2446, 5′-ATACACTGTGCCAAATGCTCCC-3′; 19S:2473–2483, 5′-TCTCTGGATCCCAGAAGGTGA-3′ and 19A:2569–2549, 5′-GTCAAGGATTTCTTTGTTGGC; 20S:2571–2591, 5′-AGCCTATGTGATGGCTAGTG-3′ and 20A:2750–2731, 5′-CAATCTGCACACACCAGTTG-3′; 21S:2757–2778, 5′-GCATGAACTACCTGGAAGATCG-3′ and 21A:2904–2882, 5′-CCCTCGGCATGATATTCTTTCTC-3′. These primers are equipped with the M13-primer sequence to facilitate sequencing. PCR was performed according to the manufacturer’s protocol. The PCR products were purified using the BigDye XTerminator^®^ Purification kit (Applied Biosystems) to remove unconsumed dNTPs and primers, and 1.5 μl aliquots were then directly sequenced using the M13-sequencing primers and BigDye Terminator^®^ v3.1 cycle sequencing kit in an ABI 3130 genetic analyzer (Applied Biosystems).

## Results

### Observational findings

The observational finding between TG mice and control mice included epilation. All of the 15 TG mice sporadically lost ~50% of their body hair, while 8 control mice lost no hair and 2 control mice had minimally sporadic hair loss. There was no statistical difference in body weight between the TG (26.7±1.10 g) and control mice (27.0±1.05 g). Confirmation of K19/JCV T-antigen^+/+^ TG mice was accomplished using real-time PCR targeting for the JCV T-antigen just before the mice were euthanized.

### Histologic and IHC findings

**Figure 1:**
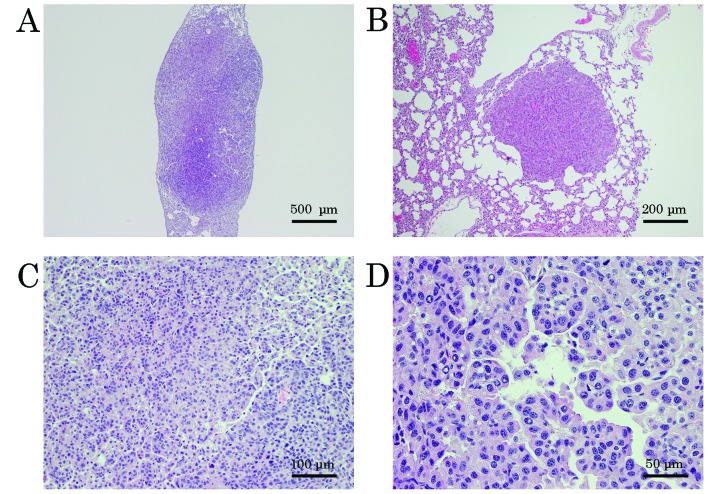
Histologic features in the pulmonary tumors of the TG mice. (A and B) A clear boundary was noted between the tumor and normal lung tissues. (C) Pulmonary tumors were composed of small tubular, solid arrangement consisting of small irregular and atypical nuclear cells. (D) Some of the tumor cells resembled hyperplastic bronchial cells. TG, transgenic.

**Figure 2:**
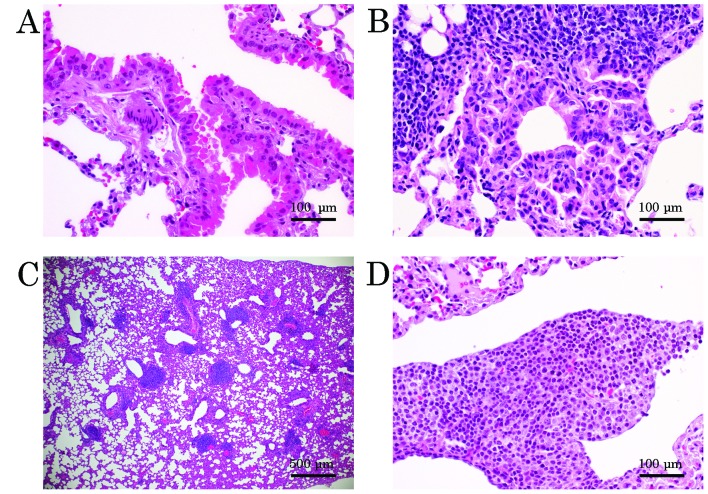
Histologic features of the non-neoplastic tumors of the TG mice. (A) Bronchial hyperplasia was more prominent in the TG mice than that in the control mice. (B) Some bronchial hyperplasia resembled pulmonary tumors, but were characterized as bronchial hyperplasia due to the small size and mild atypia. (C) A clear tendency was found toward sporadic aggregation of lymphocytes in the TG mice, (D) some of which showed monotone features and mild atypia. TG, transgenic.

Two pulmonary tumors were identified in the TG mice (13.3% of TG mice), while no tumors were found in any other organs examined including the esophagus, stomach, small intestine, large intestine, heart, liver, pancreas, kidney, spleen and brain. One pulmonary tumor occupied over half of the left lung without pulmonary metastasis in a male TG mouse. Histologically, there was a clear boundary between the tumor and normal lung tissue and a small tubular, solid arrangement consisting of small irregular and atypical nuclear cells (Fig. 1A and C). Some of the tumor cells morphologically resembled hyperplastic bronchial cells (Fig. 1D). Another pulmonary small tumor without pulmonary metastasis was found in the left lung of a female TG mouse. The histologic examination of this tumor showed a clear boundary between the tumor and normal lung tissue and a small, tubular arrangement consisting of round- to oval-shaped and mildly atypical nuclear cells (Fig. 1B). Bronchial hyperplasia was more prominent in TG mice than in control mice (Fig. 2A). Some bronchial hyperplasia morphologically resembled a pulmonary tumor, but was characterized as bronchial hyperplasia due to the small size and mild atypia (Fig. 2B).

There was a clear tendency toward sporadic aggregation of lymphocytes in the TG mice (Fig. 2C), some of which showed monotone features and mild atypia (Fig. 2D). This finding was not observed in the control mice.

**Figure 3:**
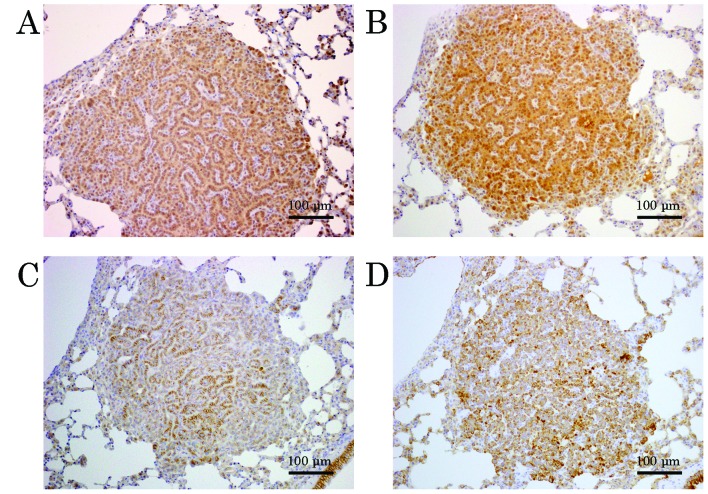
Comparison of IHC staining patterns for JCV T-antigen, p53, β-catenin and CK-19 in the pulmonary tumors. Pulmonary tumor cells demonstrated expression of (A) JCV T-antigen and (B) p53. Expression of (C) β-catenin was found to be membranous and (D) CK-19 was found to be cytoplasmic. IHC, immunohistochemistry; JCV, JC virus.

**Figure 4:**
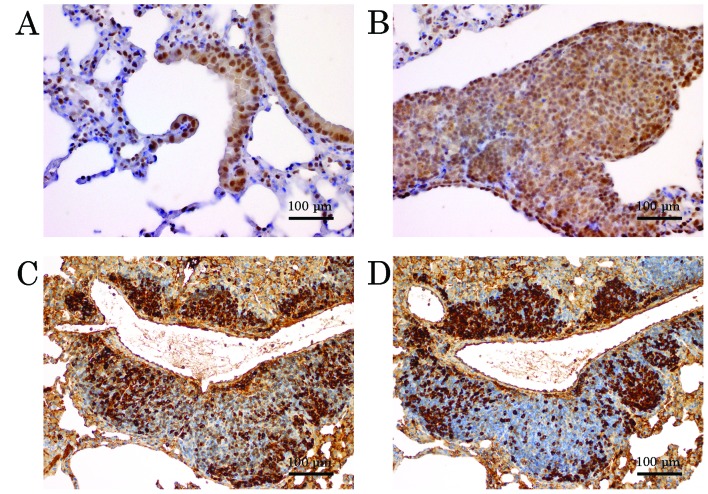
IHC features in bronchial epithelium and lymphoid aggregates. (A) Bronchial epithelium exhibited expression of the JCV T-antigen. (B) Scattered aggregations of lymphocytes were usually positive for JCV T-antigen, while lymphocytes demonstrated polyclonal immunohistochemical reactivity. (C) CD3(+) lymphocytes and (D) CD79a(+) lymphocytes. IHC, immunohistochemistry; JCV, JC virus.

IHC revealed that the pulmonary tumors were positive for JCV T-antigen primarily in the nuclei (Fig. 3A). p53 was also noted primarily in the nuclei (Fig. 3B), with membranous β-catenin (Fig. 3C) and cytoplasmic CK-19 (Fig. 3D). Insulin receptor substrate-1 (IRS-1), used as an alternate for insulin-like growth factor receptor (IGFR), was negative as determined by IHC. Tumor cells were CK7(+) and CK20(−). Similar IHC findings were noted in the bronchial epithelium. As shown in Fig. 4A, positivity for JCV T-antigen was noted in the bronchial epithelium.

Scattered aggregations of lymphocytes as shown in Fig. 2C were usually positive for the JCV T-antigen (Fig. 4B) and p53. However, lymphocytes demonstrated polyclonal immunohistochemical reactivity. Some lymphocytes represented T lymphocyte clones, and were positive for CD3 and CD5, as well as balanced CD4 and CD8 positivity. Other lymphocytes represented B lymphocyte clones, and were positive for CD20 and CD79a and, to a lesser degree, CD10. Fig. 4C illustrates CD3(+) lymphocytes and Fig. 4D illustrates CD79a(+) lymphocytes.

### Mutation analyses of K-ras and EGFR

**Figure 5:**
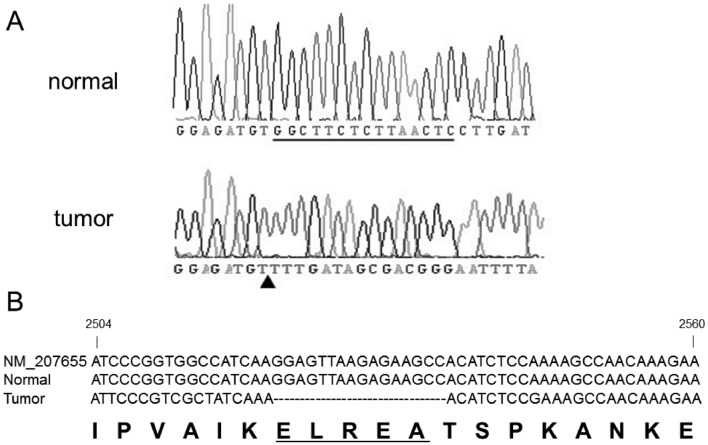
Detection of a 15-bp deletion in exon 19 of the EGFR gene in a tumor. (A) Electropherograms of the sequencing (bottom strand) of lung tissues. Underlined sequence in the normal benign lung tissue was deleted in the tumor at the position indicated by an arrowhead. (B) Translation of the sequence around the deletion showing an in-frame deletion of the amino acids ELREA. NM_207655, EGFR mRNA sequence in Genebank for the reference. EGFR, epidermal growth factor receptor.

No mutations in *K-ras* were observed. An *EGFR* mutation was detected in the in-frame exon 19 deletion that eliminates the 5 amino acids glucine-leucine-arginine-glucine-alanine ELREA (del E841-A845). No abnormalities were detected in the corresponding normal benign lung tissue (Fig. 5).

## Discussion

This is the first report showing that JCV may be associated with epithelial tumorigenesis in experimental animals. The possibility that spontaneous pulmonary tumors should arise in TG mice is very low in this case, as the back strain of this TG mouse, C57BL/6J, is almost completely resistant to spontaneous pulmonary tumors (19). We did not detect any tumors in other organs; most notably no tumors were detected in the brain. We also did not detect any intra-pulmonary or distant metastasis.

Conventional mouse models for pulmonary cancer almost exclusively give rise to solid, papillary or bronchiolo-alveolar adenocarcinomas. These single or mixed tumor types retain marker expression profiles characteristic for both type II alveolar as well as Clara cells, and therefore both cell types can serve as hypothetical cells of origin for mouse non-small cell lung cancer (19). Similar experiments demonstrated that mice expressing SV40 large T-antigen from the Clara cell-specific CC10 promoter or alveolar type II cell-specific promoter developed early multifocal bronchial alveolar hyperplasia followed by mixed solid and papillary adenocarcinomas to which the mice succumbed by 4–5 months of age (20–22). Ectopic expression of the HPV-16 E6/E7 transgene under the control of the keratine-5 promoter resulted in lung adenocarcinoma after ~6 months of age (23).

The JCV T-antigen can trigger progression of the cell cycle into the S phase in host cells (3,24) and interacts with p53, resulting in interference with p53 (25) and allowing additional genetic damage, representing a step forward in tumorigenesis (26,27). In the present study, p53 was expressed exclusively in pulmonary tumors and bronchial epithelium. Although we could not determine whether this p53 was wild-type or the mutated type, we hypothesized that p53 may be linked to pulmonary tumorigenesis induced by the JCV T-antigen. The JCV T-antigen can also interact with IRS-1, a major protein of the insulin-like growth factor I receptor (IGF-IR) signaling pathway, which is activated and translocated to the nucleus in the presence of the JCV T antigen (28). Activated IRS-1 is an adaptor in the cell response to insulin, activating phosphatidylinositol 3-kinase (PI3K), which is implicated in cell survival (29) and proliferation signals (30). The JCV T-antigen can also inhibit homologous recombination-directed DNA repair (HRR) causing DNA damage, mechanistically by its interaction with IRS-1 (31), which also interacts with Rad51 at locations of damaged DNA (32), and thus may contribute to the generation of genetic instability in cells containing JCV (33). In this study, expression of IRS-1 was not found in any type of cells, and therefore we could not find evidence of this mechanism.

The JCV T-antigen also contributes to the stabilization of β-catenin by a novel mechanism mediated by the small GTPase Rac1 (34). β-catenin is an integral component of the Wnt signaling pathway, and stabilization of β-catenin is associated with increased transcription of genes that regulate cellular proliferation, e.g., c-myc and cyclin D1 (8). In our study, expression of β-catenin was restricted to the membrane of tumors and bronchial epithelial cells, but was not expressed in the cytoplasm or nucleus, which led us to hypothesize that expression of β-catenin merely played the role of an adhesion molecule in these cells.

Another mechanism by which JCV may contribute to pulmonary tumorigenesis is similar in some aspects to the role of human papilloma virus (HPV) in cervical carcinoma (35). Viral DNA integration has been only partially addressed in cervical cancer associated with HPV (36). The chromosomal locations of HPV viral integration sites coincide with those of fragile chromosome sites (37) and translocation break-points which have been already detected in other types of carcinomas (38). If the JCV infection is persistent, then there is a high probability that JCV DNA may integrate into the host cell genome, while some of the viral DNA remains similarly to what occurs with HPV in cervical carcinoma (37).

In mouse models of chemical pulmonary tumorigenesis, in which the type of tumor is adenoma or adenocarcinoma, *K-ras* mutations are frequently observed, whereas genetic alteration of *EGFR* is generally rare (38). In the present study, only one case of bronchial epithelial hyperplasia was examined. In this case, an *EGFR* mutation was detected, while a *K-ras* mutation was not detected. While it is unclear how mutations of *EGFR* and other molecular alterations are related to the mechanisms involved in pulmonary tumorigenesis in mice, both genetic and epigenetic factors are known to be important for tumorigenesis (39). In this case, it is impossible to resolve the mechanism of the phenomenon based on only one tumor.

In this study, we found that some lymphocytes were positive for both the JCV T-antigen and p53 in the nuclei. However, these lymphocytes were not found to be monoclonal by IHC. The hypothesis suggesting that lymphocytes mediate JCV infection was reported in a previous study (40). Lymphocytes may have tolerance for JCV activation, leading to positivity for both JCV T-antigen and p53.
